# Explaining the reasons for the divorce of young couples in Western Iran: A qualitative Study from the Beneficiary's point of view

**DOI:** 10.1371/journal.pone.0354355

**Published:** 2026-07-30

**Authors:** Javad Yoosefi Lebni, Seyed Fahim Irandoost, Mandana Saki, Bahar Khosravi, Ahmad Ahmadi

**Affiliations:** 1 Social Determinants of Health Research Center, Lorestan University of Medical Sciences, Khorramabad, Iran; 2 Department of Community Medicine, School of Medicine, Urmia University of Medical Sciences, Urmia, Iran; 3 Social Determinants of Health Research Center, School of Nursing and Midwifery, Lorestan University of Medical Sciences, Khorramabad, Iran; 4 Social Determinants of Health Research Center, Lorestan University of Medical Sciences, Khorramabad, Iran; 5 PhD student in Educational Technology, Faculty of Psychology and Educational Sciences, Allameh Tabataba’i University, Tehran, Iran; Tarbiat Modares University Faculty of Medical Sciences, IRAN, ISLAMIC REPUBLIC OF

## Abstract

Divorce is an important social issue that has destructive consequences for both the individual and the family. It is a multi-dimensional and complex phenomenon that occurs under the influence of various factors, so this research was conducted to explain the reasons for divorce among young couples in the West of Iran with a qualitative approach. This research was carried out with a qualitative approach and conventional content analysis. Purposeful and snowball sampling were used to identify the participants. Semi-structured interviews were used to collect data and continued until theoretical saturation was reached. In this research, 16 key informants and 32 main participants were present. For data analysis, Graneheim and Lundman method was used. Also, to increase the quality of the research, Guba and Lincoln's four criteria were observed. 212 primary codes, 24 subcategories, and 3 categories came from the analysis of the interviews. Categories and sub-categories include 1- individual factors (sexual problems, mental-psychological disorders, spouse's behavioral characteristics, individualism, physical appearance problems, Formation of a positive understanding of divorce consequences, having defective relationships before marriage, drug use), 2- socio-cultural factors (de-tabooing of divorce, women-oriented social changes, media and social networks, lack of a suitable support system, inappropriate spouse choosing, economic challenges, modeling of divorce), 3- family factors (job's impact on family life, improper communication with spouse's family, accumulation of negative feelings in marital life, issues related to children, cheating on spouse, inefficient relationships, heterogeneity of lifestyle, violence, inability to manage challenges). The results indicate that divorce is a complex, multi-dimensional phenomenon influenced by individual, social, cultural, and family factors. Prevention requires multi-level interventions, including educating couples on sexual issues and communication skills, promoting responsible marital roles, preventing superficial marriages, reducing stigma around seeking psychological help, managing personality and lifestyle differences, guiding families on conflict resolution, and providing access to counseling and mental health services before and after marriage.

## Introduction

In terms of age, the family is the most primitive and in terms of its scope, the most universal social institution that is formed by marriage and has existed since the beginning of mankind, without it the central core of any society would not exist [1]. Experts believe that the family is one of the first public and global systems necessary to meet the vital and emotional needs of humans and for the survival of society. proper relationships in society are formed based on proper relationships in the family and the more suitable the relationships within the family are, the more stable and strong the family and, consequently, the society are [2]. The family has a special and important role in all social institutions, organizations, and groups. A society cannot claim to be healthy without healthy families, and none of the social harms have emerged without affecting the family [3,4].

Thus, since the family is the most important institution of society and the origin of a healthy society is a healthy family, any damage and deterioration to this institution can have very destructive consequences for society. Marriage is the first and most important stage in the life cycle of a family, and success in other stages of life depends on its success [5,6]. However, in some cases, the marriage may cause couples to face challenges resulting in divorce. Divorce is one of the most important family injuries, which is the emotional and physical process of separation of a couple that happens over time and through different stages [7,8]. Globally, divorce has been on the rise over time [9]. A study conducted among 33 sub-Saharan African countries reported that 25% of first marriages end in divorce [10]. Iranian society also like many other countries has faced a growing number of legal divorces in the last few decades [8,11]. Although Iran is an Islamic country, and in Islam, maintaining a marital life and avoiding divorce has been emphasized, it has the highest growth rate of divorce among Islamic and North African countries (7). Divorce in Iran has been on the rise for the past two decades. In 2015, there were 12 divorces per hundred marriages, which reached 33 in 2019 and 47 in the first two months of 2022 [12].

Previous studies have shown that divorce can lead to problems and harm such as physical and mental disorders [13,14], self-immolation, suicide, addiction, sexual deviations, and crime and delinquency of children, etc. [15–21]. It has been reported in various studies that divorce can be caused by factors such as addiction, violence, sexual and emotional dissatisfaction, family interference, financial problems, cultural differences, individualism, etc. [22–24].

In a research conducted in Semnan, Iran in 2022, it was also reported that violence, the absence of a culture of dialogue between families, cultural differences with spouses, lack of responsibility for family life, and economic problems were the most important reasons among divorce applicants [25]. In another study conducted by Osafo et al. in Ghana in 2021, they stated that cheating, abuse, financial support, intimacy, financial problems, third-party influence, and lack of respect for the spouse are the most important reasons for the occurrence of divorce [26]. In research they conducted in Pakistan, Qamar et al. (2021) mentioned the interference of others, lack of mutual understanding, financial exploitation, and an agonizing environment as the reasons for divorce [27].

Understanding the cultural context of divorce in Iran is essential, as the meaning, causes, and consequences of divorce are deeply embedded in cultural norms and values. Iranian society, while undergoing rapid social changes, still retains strong traditional and religious influences, particularly in smaller cities and rural areas. In the western region of Iran, including the province of Lorestan where this study was conducted, family structures are predominantly traditional, with strong emphasis on collective family honor, gender roles, and community surveillance. In such contexts, divorce has historically been stigmatized and considered a last resort. However, recent decades have witnessed significant cultural shifts, including increased access to education and employment for women, greater exposure to global media and social networks, and changing attitudes toward marriage and divorce. These changes have gradually reduced the taboo surrounding divorce, especially among younger generations. Yet, traditional cultural expectations regarding women's roles, family mediation, and the importance of maintaining family reputation continue to influence how divorce is perceived and experienced. Therefore, examining divorce in this region requires a culturally sensitive approach that takes into account both persistent traditional values and emerging modern attitudes.

Despite the importance of this topic, several research gaps exist. First, most studies conducted on divorce in Iran have been quantitative, focusing on statistical relationships rather than the deep, lived experiences of individuals. Second, the few qualitative studies available have predominantly investigated only women's perspectives, while the views of men, families, and key informants such as family counselors, lawyers, sociologists, and psychologists, who can provide valuable and comprehensive insights into the phenomenon, have been largely overlooked. Third, the causes of divorce are highly context specific, and findings from other countries or even from other ethnic groups within Iran cannot be generalized to the unique cultural, social, and economic context of western Iran, particularly the cities of Borujerd, Dorud, and Delfan, where no comprehensive qualitative study has yet explored the reasons for divorce among young couples. Given that divorce rates have been rising rapidly in Iran, and considering the destructive consequences of divorce for individuals, families, and society, it is of special importance and necessity to identify and explain the specific factors contributing to divorce in this understudied region. Therefore, the present study aims to explain the reasons for divorce among young couples in western Iran using a qualitative approach from the perspective of all beneficiaries, including divorced individuals, those on the verge of divorce, and key informants.

## Methods

### Design

This research was conducted with a qualitative approach and using the conventional content analysis method. The knowledge produced in the content analysis method is based on the unique experiences of the participants and from the real data. In qualitative content analysis, the goal is to group and classify the information obtained from the interviews and observation protocols. In this method, information is collected directly from the participants, and codes and classes are extracted from raw data by inductive method [28]. The reasons for choosing this method are the lack of comprehensive information about the reasons for the divorce of young couples in the cities of Borujerd, Durud, and Delfan, limitations of the quantitative and qualitative research conducted in this field, the specificity of the reasons for divorce to the culture, manners, and customs of different regions and the need to investigate this phenomenon in the social and cultural context of Lorestan.

### Participants

This research was carried out in the cities of Borujard, Durood, and Delfan in the west of Iran. The participants in the research are a diverse range of different groups, the first group included young men and women who have been divorced in the last 4 years, and the second group was young men and women who are on the verge of divorce and have gone to court. The third group was key informants that included a wide range of experts with experience and knowledge on the subject of divorce. These people were family counselors, family lawyers, sociologists, psychologists, and people who worked in the courts in the field of divorce.

The criterion for entering the study was to have experience of divorce in the last 4 years or to go to court for divorce at present and to be less than 35 years of age for both; for the key informants, the criterion was to have experience and knowledge of the issue of divorce. The exclusion criterion was not completing the interview process and not allowing audio recording. In this study, “young couples” refers to individuals under 35 years of age. All participants in the study were between 20 and 34 years old, and no individual aged 35 or older was included in the study.

Participants were initially selected through purposive sampling and then snowball sampling. To do that, by referring to family courts, young couples who were on the verge of divorce or couples who have divorced in the past 4 years were identified, and by meeting them and obtaining their permission, interviews were conducted. Then to increase the number of interviews and to reach saturation, at the end of each interview, the participants were asked to introduce other people, whom they knew and who met the criteria for the study, to the researcher. 20 of the main participants were selected through purposive sampling and 12 through snowball sampling. Considering the key informants, the researcher went to the family courts and interviewed lawyers, family counselors, and other people. Also, interviews were conducted with psychologists or sociologists who were active in the province and had good knowledge and information in the field of divorce. In general, 16 key informants with various fields and specialties were selected to participate in the research. It should be noted that in the selection of participants, the strategy of diversity in sampling was also considered, that is, it was tried to select participants who have the most diversity in terms of demographic characteristics and provide the most help for understanding the phenomenon.

### Data collection

Data collection was conducted between 05/06/2024 and 18/08/2024 using semi-structured face-to-face interviews with eligible participants. Before the interviews, the researcher presented a brief biography of himself first, then explained the general purpose of the research and the method of data collection and dissemination to the participants, and after the consent of the participants, the interviews began. At first, some demographic questions including age, education, and occupation were asked, and then the interview started with a general question like “What made you think of divorce?” or “Why did you get divorced?.” Then other questions were asked to analyze the phenomenon, such as what are your most important reasons for divorce? What role did the family play? What was the role of the society and the surrounding people? Also, regarding the key informants, questions such as, in your opinion, what causes couples to divorce? What are the most important reasons for divorce in the target society from your point of view? Were asked.

The time and place of the interviews were determined by the participants. During the interview, no one else was present except the researcher and the participant. The interviews were conducted by an expert with a lot of experience in the field of qualitative research. The duration of the interview was different for each of the participants according to their information and experiences. The average duration of the interviews was 62 minutes. Data analysis started after the first interview and every interview that took place was immediately analyzed and coded so that the codes were raised in the form of questions in subsequent interviews and were constantly reviewed and compared. Data collection and coding continued until reaching theoretical saturation that is until no new reason for divorce was presented and all the codes were repeated. Theoretical saturation in qualitative research happens when all codes become repetitive and the continuation of interviews does not add anything new to the research.

### Data analysis

Data classification was done with MAXQDA 2020 software and its analysis was done using the Graneheim and Lundman method [28]. The data analysis was done by the first author and the responsible author of the article and was finalized with the supervision and consensus of all the authors of the article. Thus, in the first stage, after conducting each interview, the interviews were immediately typed and saved in Word 2013 software. In the second stage, the text of the interviews was read and reviewed several times by the researchers to reach a general understanding of the text of the interviews. In the third step, all the texts were read word by word and very carefully and the codes were extracted. In the fourth step, the codes that were similar in terms of content and meaning were placed in the same category and their relationship was determined. In the fifth step, the data were placed in the main classifications, which were more general and abstract than the previous classification, and the themes were extracted.

### Trustworthiness and robustness of the study

In order to increase the accuracy of the research, Guba and Lincoln's criteria were observed [29]. During the research, it was tried to select participants with different demographic characteristics (in terms of age, economic status, education, etc.) in order to have maximum diversity. In addition to the fact that at the end of each interview, the researcher expressed his understanding of the participant's speech in general and in summary to see if he understood his speech correctly or not, and he agreed with them. Also, at the end of the research, the findings were given to several participants to see if the researcher had expressed their opinions and experiences correctly or not, which was approved by all of them with minor modifications, in this way, the credibility of the data was obtained. To obtain confirmability, the main researchers observed impartiality in the entire research process. Also, during the coding and analysis of the data, all the authors of the article were active and engaged in discussions, and eventually, the categories and subcategories were approved by all of them. Then the table of research findings was given to 5 researchers familiar with the topic of divorce to express their views and if necessary, make changes, which were approved by making partial changes in the naming of the subcategories. To control the dependability, the researchers also wrote the entire research process explicitly and clearly and also recorded the interviews and notes immediately after the interviews and all the members of the research team supervised the process of conducting the study. To increase the transferability, all the study steps, the path, and the field of the study were carefully described, and the direct quotes of the participants were included in the research a lot. Also, the table of categories, subcategories, codes, and the entire section of findings was sent to 6 People who met the inclusion criteria to enter the study but did not participate in the research to evaluate whether the findings of the research were in accordance with their experiences and feelings, which was confirmed by them.

### Ethical considerations

The study was approved by the Ethics Committee of Lorestan University of Medical Sciences (IR.LUMS.REC.1403.038). All methods were performed in accordance with the relevant guidelines and regulations of Lorestan University of Medical Sciences. To comply with ethics in the research, written consent was obtained from all the participants, and at the beginning of each interview, the researcher briefly explained the objectives of the research as well as the conditions of participation in the research and how to interview the participants and told them that participation in the research was completely optional and anytime, they wanted, they could withdraw from the interview or choose another time and place. At the end, the method of reporting the findings was explained to the participants and they were assured that the recorded file will only be used in accordance with the objectives of the research and no one else will have access to it, and after typing the interviews, the recorded files should be completely deleted, and in the publication of the findings, no names or addresses of them should be presented so that they cannot be identified.

## Results

In this research, 16 key informants (Table 1) and 32 main participants (Table 2) were present. Also, from the analysis of the interviews, 212 initial codes, 24 subcategories and 3 categories were obtained (Table 3), which are mentioned below along with each of the following categories and direct quotes of the participants.

**Table 1:** Demographic characteristics of key informants participating in the study.

Marital status	Job	Education	Age	Gender	NO.
Married	Lawyer	MA in law	41	Female	1
Single	family counsellor	PhD in psychology	31	Female	2
Married	Lawyer	PhD in law	41	Male	3
Married	Teacher (divorcee's father)	BS in chemistry	55	Male	4
Single	Lawyer	MA in law	39	Male	5
Married	Court employee	BA of social work	40	Female	6
Single	employee of justice administration	BA of family studies	29	Male	7
Married	University professor	PhD in sociology	48	Male	8
Single	University student	PhD in demography	37	Female	9
Single	Family counsellor	PhD in psychology	32	Male	10
Married	Court employee	MA in education sciences	37	Female	11
Married	Researcher in women studies	PhD in sociology	49	Male	12
Married	Housewife (divorcee’ mother)	Elementary	48	Female	13
Married	Farmer (divorcee’ father)	High school diploma	54	Male	14
Married	Employee (divorcee's mother)	MA in women studies	46	Female	15
Married	Shopkeeper (divorcee's father)	Diploma of junior high school	58	Male	16

**Table 2:** Demographic information of participants.

Frequency	Group	Variable
4	Under 20	Age
9	20 - 25	
11	26 - 30	
8	31-34	
14	Male	Gender
18	Female	
3	Illiterate	Education
4	Elementary & junior high school	
8	High school & Associate degree	
10	Bachelor	
7	MA and PhD	
17	Urban	Residence place
15	Rural	
7	Under 1 year	Length of marital life
15	1–5 years	
8	6–10 years	
2	Over 10 years	
10	Unemployed	Job
14	Self-employed	
8	Clerk	
10	No children	The number of kids
17	One	
5	Two or more	

**Table 3:** Codes, categories and subcategories obtained from interview analysis.

categories	subcategories	Codes
Individual factors	Sexual problems	Not being satisfied by the spouse, sexual indulgence, having wrong sexual ideas and expectations from the spouse, premature ejaculation, weak sexual relations
	Mental-psychological disorders	having bipolar disorder, severe obsession, depression, anxiety disorders, schizophrenia, paranoid personality disorder
	Spouse's behavioral characteristics	Nervousness, pessimism towards spouse, lack of personal independence, weak ability to express love, being stingy, controlling spouse, being fanatical
	Individualism	Indifference towards children's future, welfare seeking, low dependence on wife and children, irresponsibility towards family, not caring about family issues, ignoring spouse and having too much contact with single friends.
	Physical appearance problems	Infertility, physical disability, one of the couple being sick, being fat, not having a beautiful appearance
	Formation of a positive understanding of divorce consequences	Divorce means escaping from an exhausting life, divorce means rebirth, looking at divorce as an opportunity to build a life you want, divorce means finding yourself, divorce means getting rid of the limitations of married life, earning income through dowry
	Having defective relationships before marriage	Multiple emotional relationships before marriage, staying in past emotional relationships, seeking sexual diversity before marriage, having unconventional sexual relationships before marriage, multiple failed relationships before marriage
	Drug use	Addiction to industrial and traditional drugs, consumption of alcoholic beverages, consumption of psychedelic pills
Socio-cultural factors	De-tabooing of divorce	Breaking the taboo of divorce in society, normalizing divorce in society, changing society's attitude towards divorced people, the availability of better marriage opportunities
	Women-oriented social changes	Changing the socio-economic status of women in the society, making women more aware of their rights, the desire for more independence among women
	Media and social networks	Addiction to social networks, comparing one's life with bloggers and celebrities on social networks, dispute over how to use and behave in social networks, spending a lot of time on satellite networks
	Lack of a suitable support system	Lack of suitable counseling centers and limited access to them to prevent divorce, incomplete process of courts to prevent divorce, incomplete support from families to continue living, ignoring traditional support networks to establish reconciliation and resolve marital conflict, not seeking advice from capable people
	Inappropriate spouse choosing	early marriage, forced marriage, having a big age difference with the spouse, not considering the cultural differences of the couple's families, not considering the class distance of the couple's families, emphasizing too much on having a certain standard on the part of the spouse and ignoring other criteria such as wealth and being a doctor, not knowing and not researching properly about the spouse's family before marriage, an inappropriate motivation for marriage
	Economic challenges	Financial problems, unemployment, having a hard and low-income job, getting tired of renting, inflation, economic shock, loss of capital, loss of capital due to improper management of income and expenses, separation of spouses’ income, financial competition
	Modeling of divorce	Modeling from friends, modeling from other family members and relatives, modeling from cinema and television
Family factors	Job's impact on family life	Dissatisfaction with the spouse's job, dissatisfaction with the spouse's working hours, dissatisfaction with the spouse's work environment, considering the spouse's work environment to be unsafe, having many work shifts and not being at home, living apart due to work conditions
	Improper communication with spouse's family	Disrespecting the spouse's family, insulting the spouse's family, not participating in the important ceremonies of the spouse's family, lack of proper and stable communication with the spouse's family, Interference of the spouse's family
	Accumulation of negative feelings in marital life	feeling disappointed of the future of married life, the feeling of fear of the future and abandonment, the feeling of the unfairness of the married life, the feeling of the destruction of dreams and interests in the married life, the feeling of superiority over the spouse
	Issues related to children	Not having a child, having a single child, having a disabled child, not having a son
	Cheating on spouse	Not being faithful to each other, having sex outside the family framework, having an emotional relationship with someone other than your spouse, having a virtual emotional relationship with someone other than your spouse
	Inefficient relationships	Emotional divorce, not talking with each other, not traveling, not creating sweet moments together, not understanding each other, secrecy, telling lies, Not caring about the role of being a spouse
	Heterogeneity of lifestyle	Lack of mutual understanding in religious beliefs and behaviors, lack of mutual understanding in clothing styles and social behaviors, lack of mutual understanding in life priorities, lack of mutual understanding in how to raise children, lack of mutual understanding in choosing the place of living, lack of mutual understanding in lifestyle and how to spend free time
	Violence	Beating, insulting and humiliating, sexual violence, humiliating the spouse in front of their families
	Inability to manage challenges	Constant fights and arguments, revenge, involving the family in a fight, pertinacity and stubbornness, hasty decision to break up

1. **Individual factors**

The first category that was obtained from the data was the individual factors that shape divorce, which included things that were related to a person's appearance and personality characteristics, as well as individual perceptions and behaviors that affected married life and it would lead to divorce.

### Sexual problems

One of the reasons for divorce was sexual problems such as premature ejaculation and having poor sex performance that one of the couples had, which caused the dissatisfaction of the other party and ultimately led to divorce. Some of the participants stated that they were not satisfied in the sexual relationship between husband and wife and their spouse could not satisfy them. In some cases, some of the participants stated that their spouses had wrong expectations and perceptions about sex, and this caused problems and sexual dissatisfaction. Also, some of the participants stated that their spouses wanted an unconventional type of sexual relationship, such as group sex. They wanted to continue this type of relationship and to involve their spouse in this relationship so that the spouse's opposition had caused the divorce. Part of these expectations and misconceptions about sexual relationship was caused by watching porn movies; due to lack of knowledge, they compared their own sexual relationship with Porn actors and since they could not establish such a sexual relationship, sexual dissatisfaction has been formed.

“My wife used to give me a cold shoulder during sex and I was not satisfied with it at all. I tolerated it at first, but later it was difficult for me” (29-year-old man, master's degree)

“My husband had strange expectations in sex. He was very hot and wanted us to have sex several times a day. She also wanted us to be like in sex movies, which I couldn't do. That's why we always argued” (33-year-old woman, bachelor's degree).

“My husband had premature ejaculation. He was my husband for two years. I couldn't enjoy sex even once. I got so upset. That was one of the reasons why I broke up with him” (22-year-old woman, diploma)

“One of the main problems that causes my clients to divorce is sexual dissatisfaction, which causes betrayal and other injuries that ultimately lead to divorce” (31-year-old woman, family counselor).

“My wife got crazy, he was a dishonorable bum, and he told me that he would like to have group sex with his friend and his wife.” (25-year-old woman, bachelor’ degree)

### Mental-psychological disorders

Another reason for couples’ separation was having mental disorders. Bipolar disorder, severe obsession, schizophrenia and paranoid personality disorder were among the mental disorders that made marital life difficult. These mental problems can be treated to some extent by counseling and going to a psychoanalyst, but due to the taboos that existed about going to a psychologist, the sick person did not go to these treatment centers, and this problem caused the aggravation of the disease and disrupted the family.

“Some of the couples who come to me have mental disorders, the most common of these disorders include bipolar disorder, severe obsession, schizophrenia, and paranoid personality disorder” (32-year-old man, family counselor)

“My wife had very obsessive behaviors, she cared a lot about cleanliness, she started fights over the smallest issue, I didn't feel comfortable at home, I always liked her to be in her father's house” (30-year-old man, diploma)

### Spouse’s behavioral characteristics

Each of the couples have certain behaviors that caused problems in their family life. One of these characteristics was the weakness in expressing love, which could be rooted in the cultural beliefs formed in the studied areas, which causes couples to not be able to express love properly, because in traditional parts of society, expressing love is unpleasant for women and may lead to the formation of the belief that she is shameless and has no decency. It is also the same for men that there is this idea that men should be strong and stiff and should not express their love too much. Of course, these thoughts and beliefs are becoming weaker day by day, but they still exist in the traditional parts of society. Another one of the most important behaviors was pessimism and suspicion towards the spouse, which caused severe control and dissatisfaction of the other party. Dependence and lack of personal independence was another behavioral characteristic that affected married life. This dependence and lack of personal independence was observed in both genders; it was more common in people who were younger. Miserliness was one of the other behavioral characteristics that caused divorce, of course, it must be said that divorce is a complex phenomenon and each of these factors alone cannot fully explain it, but in the life of two people, a set of factors go hand in hand and leads to separation.

“My husband was very nervous, he was always angry and he could do anything when he was angry. I was afraid of him, that's why I divorced” (19-year-old woman, junior high school)

“My wife was sick, she thought I was cheating on her, she checked my phone every day when I went home.” (33-year-old man, associate degree)

“My mother-in-law and sister-in-law had a lot of influence on my wife, she did whatever they said and was very dependent on them; this affected our life” (21-year-old man, diploma)

“My husband was stingy, his income was very good, but it killed him to buy a kilo of fruit for the house, I always had a problem with him buying clothes for the children, he said he didn't want me to buy so many clothes for them, and I didn't want such a life anymore” (30-year-old woman, associate degree)

“My wife didn't know how to express her love to me at all. She said it was rude. We talked a lot about this issue, but she thought that if she expressed her love to me, I would think badly of her. She was very traditional and grew up in a very traditional family, unlike me. (34-year-old man, PhD)

### Individualism

Another reason for divorce was individualism, which was seen in some couples. Some of the participants stated that their spouse had no commitment towards them and their children and only thought about their own pleasure and having fun. This individualism had caused a man or woman to not have much commitment to the family and to be less present in the family space so that they spend most of their time with their single friends or pursue their interests and hobbies and do not care much about family problems.

“My wife used to allocate most of her time to the hairdressing salon, she didn't think of me at all, she only cared about what new makeup model or what new coat was on the market” (24-year-old man, bachelor's degree)

“My husband didn't think about me and my daughter at all. He left us alone for many days and went to have fun with his friends. Once my daughter got sick, she was in the hospital, but my husband went to northern Iran (seaside) with his friends the next day” (21-year-old woman, diploma)

### Physical appearance problems

Among the other reasons for divorce that were stated by the participants were physical-appearance problems, which included problems such as infertility, physical disability, one of the spouses being sick, being fat and not being good-looking. Due to the importance of fertility issue in Iranian culture, infertile people are more exposed to its consequences, of course, these consequences are more for women, because in Iran, most of the popular culture considers infertility as a female issue. Impairment was one of the other reasons for divorce, and it happened mostly in cases where this impairment was caused after marriage and due to accidents or occupational accidents. Some of the participants also stated that after they were diagnosed with a certain disease, their spouse left them and got a divorce. Being fat and not having a beautiful appearance was one of the other reasons for divorce, which was mostly expressed by men, and they said that they separated from their wives because they were fat and did not have the necessary beauty.

“My wife couldn't have a child, that's why we separated, although my family's insistence was not without effect” (29-year-old man, bachelor's degree)

“My husband's spinal cord was severed in an accident, he couldn't do anything, I really couldn't bear that life anymore, that's why I decided to get a divorce.” (34-year-old woman, primary school)

“Appearance was very important to me, but my wife did not come to herself at all after marriage. No matter what I told her, she said that these things were past me. After giving birth to my son, who became fat, I tried very hard to convince her to come to herself more, but she would not listen to me. We had a problem with this issue and we broke up”) (26-year-old man, graduate)

### Formation of a positive understanding of divorce consequences

In traditional societies, divorce is always viewed as an ugly phenomenon, but recently in the world and Iran, people's understanding and view of this phenomenon have been accompanied by many changes, so not only this phenomenon is not considered ugly, but also, they have found a positive understanding of it. In some cases, some participants look at divorce as an escape from an exhausting life, a rebirth, and an opportunity to build a desired life, to find themselves, and to get rid of the limitations of married life. However, some woman participants looked at divorce as a tool and considered it as a way to get rich and financially independent, because by receiving dowry from their husbands, they could earn good money, although receiving dowry is a time-consuming process and they face many challenges.

“My life was like hell; every day was painful for me. I think sometimes divorce is like a miracle that can save your life” (18-year-old woman, junior high school)

“After getting divorced, it's like I was born again. If I had stayed in that life, my life would have been ruined, but now my situation is much better.” (24-year-old woman, bachelor's degree)

“Married life is full of restrictions and responsibilities, and the only thing that can help you get out of that responsibility is divorce” (22-year-old man, associate degree)

“From the very beginning, I was looking for a rich husband to spend money for me. After two years, I separated from him and took my dowry.” (34-year-old woman, diploma)

### Having defective relationships before marriage

Another reason for divorce was inappropriate relationships before marriage. Some participants were still in their emotional or sexual relationships before marriage, and this affected their married life. Of course, the type of relationship before marriage and the variety of it influenced the behavior of couples after marriage. For example, if they had many sexual and emotional relationships before marriage, it caused a kind of diversity-seeking in them, and after marriage, this diversity-seeking did not decrease and then affected their married life in such a way that resulted in separation and divorce.

“Before I got married, I experienced several love failures that took away all my spirit and feelings, so that I no longer had any feelings to spend on my husband” (34-year-old woman, master's degree)

“After marriage, I found out that my wife loved someone else before marrying me, but her family disagreed. I found out that my wife was still involved in the previous relationship, although she was not cheating, she was just following to see what her partner was doing and whether he was married or not, but such a thing messed me up. We gradually got apart because my wife didn't love me at all, her heart wasn't with me, that's why we broke up” (30-year-old man, bachelor's degree)

“My husband was one of those guys who had relationships with hundreds of people before marriage, that's why he remained the same after marriage. I thought it would be better if we got married, but it got worse.” (21-year-old woman, junior high school)

“My single life was very bad. I got involved with many boys, but they all left me. After marriage, I thought my husband would leave me one day, so I didn't feel very good.” (29-year-old woman, master's degree)

### Drug use

Another reason for divorce was drug use by one or both of the spouses, however in most cases, it was drug use among men that caused their wives to divorce them. Apart from the discussion of the financial burden that drug use has placed on the family economy, there was also the discussion of behaviors that were created following drug use and caused violence and dangerous behaviors against the wife and children. As some of the participants said that they no longer had any security due to the addiction of their spouses and were threatened and abused by their spouses every day. Violent behaviors were more among people who used industrial drugs such as crystal meth, and in most cases, the main reasons for divorce were not the problem of addiction itself, but the behaviors after drug use.

“One year after our marriage, I found out that my husband had an addiction, and I knew that continuing to live would only make me ruin my life, that's why I divorced” (18-year-old woman, junior high school)

“My husband used to use crystal meth, his behavior was strange. Sometimes he would beat me to death for things, then he would regret it, saying it was not his fault.” (33-year-old woman, primary school)

“Drug use is one of the main reasons for divorce in my clients. Drug use increases the costs of the family, and because most of them are poor, it becomes difficult to continue living. Crazy behaviors due to hangovers or industrial drug use are also effective.” (32 years old, family counselor)

1. **Socio-cultural factors**

The second category that was obtained from the data was the social and cultural factors forming divorce, which played an essential role in the occurrence of the divorce phenomenon, because these social and cultural factors somehow changed society's view on divorce and the way it reacts. Also, the change in the fabric of society caused extensive changes in the lives of individuals and families, and these changes were ultimately part of the factors that led to divorce.

### De-tabooing of divorce

Until one or two decades ago, there were traditional thought patterns about marriage and divorce in the studied societies, so many people considered divorce as an ominous phenomenon that people should refrain from doing in any way. Many cultural beliefs were formed in this field. One of these beliefs was that a girl goes to her husband's house in a white wedding dress and must return to her father's house wearing a white shroud, which indicated that divorce should not take place under any circumstances. But these beliefs and thoughts have undergone many changes in the last decade or two per the social changes that took place in society so that somehow divorce is no longer taboo and talking about it and doing it no longer face social punishments like before. This affects the tendency of women and even men to divorce. Of course, it should be noted that the social consequences of divorce are more for women than for men.

“Previously, many women and men used to preserve their lives as much as they could, but now they leave each other very easily” (54-year-old man, divorcee's father)

“My life was not good, that's why I divorced because I didn't see the need to stay in life to be bothered. Divorce is not always bad.” (19-year-old woman, diploma)

“Previously, everyone who got divorced could not get married anymore, but now it is not like that. Many people can have a proper marriage after getting divorced, society's view of divorced people has changed, now no one looks at them as criminals anymore.” (48-year-old man, PhD in sociology)

### Women-oriented social changes

In Iran, with the growth of women's education and employment, there have been many social changes in the economic and social status of women in recent decades. Women's education increased their awareness of their rights and they were no longer satisfied with continuing their lives in any way as before. It also raised their expectations from their married life. Also, women's employment made them financially independent and they could separate and continue their lives more easily than before, so they could deal with divorce more easily.

“The issue of divorce is mostly because of women. Before, they used to be beaten and didn't say anything. Now, if you say something to them, they will get a divorce. Their hands are in their own pockets [Iranian proverb: i.e., they're financially independent] and they don't have any problems anymore” (54-year-old man, divorcee's father)

“A woman who has a high education and is financially independent thinks about divorce much more easily, the things that made my wife divorce me were these two things” (33-year-old man, PhD)

“Women are not like before, they don't accept anything. You can't force them anymore, now they speak their rights as soon as you want to say something” (29-year-old man, judicial employee)

### Media and social networks

In the last two decades, there has been a great change in the access to social networks in Iran, and almost all social networks have become accessible to the general public. Because they had not received any training in this field before, this problem caused many challenges in the lives of families. Some people spend many hours of their time on social networks, as they become dependent on it, and this dependence itself will cause many problems in families. As some participants reported that the reason for divorce was their spouse's Internet addiction. Although internet addiction alone cannot be the source of divorce, it can be the cause of divorce with its consequences. Social networks gave rise to famous people who had no special art or abilities, but with the techniques they had, they were able to create a lot of popularity for themselves, so that their pages are visited by millions of people. These people, who are known as bloggers, usually expose only the exciting and enjoyable part of their lives to the public, this causes people who do not have media knowledge to compare their lives with them, and this itself can make married life confront many problems. Another thing that created a challenge in married life was the way of behavior and dress code in social networks, which was the source of many conflicts that caused separation. Of course, in most cases, it was men who could not tolerate their wives behaving against their wishes on social networks. These behaviors could be a wide range of actions from the way they dress and post pictures on their profiles, or being in different groups and talking to strangers.

“My husband was always on social networks, he didn't spend time with me and my daughter at all, he rarely went out with us” (27-year-old woman, bachelor's degree)

“Many young people, especially women, compare their lives with these celebrities and their expectations always rise and they find a lot of problems” (48-year-old man, PhD in sociology)

“These social networks have led all the young people astray; the married ones are worse. Many divorces are because of these social networks” (40-year-old woman, court clerk)

“My husband used to say that I shouldn't post my profile picture or be a member of telegram groups. He would always check my telegram. It made me nervous. We always fought over this issue” (17-year-old female, diploma)

### Lack of a suitable support system

Until a few decades ago, if there was a dispute between families, the elders would resolve it and encourage the young couple to continue their married life, but today this is no longer the case and the traditional support network has lost its importance. The elders are less willing to help and reconnect men and women. In general, in the past, most of the marriages took place within the group (intragroup marriages) and the families played a big role in marriage and the choice of the person, that is why they gave the most support to them after marriage, but today most of the marriages are out of the group (intergroup marriages) and the young people themselves often choose and the role of families has decreased, that's why families support less during tensions. However, this decrease in support can be caused by the youth's lack of attention to the words and ideas of the elders. Despite the reduction of traditional support networks, new support networks such as counseling centers have not been able to find their place. Couples who are facing problems in life are less likely to refer to these centers, and family courts do not have a proper mechanism to prevent divorce, although sometimes they send couples to counseling centers, these centers do not have a coherent process for reuniting couples and solving their problems, and in some cases, they only make the divorce process time-consuming and exhausting.

“We got married traditionally, even though I knew early on that we had problems with each other, but there was no place for me to go. The counseling centers were in the center of the province, but we were remiss and didn't go” (Man, 28 years old, master's degree)

“When couples go to the court for divorce, the courts do not have a good and defined mechanism on how to advise and guide the couple so that they do not get divorced. The only thing the court does is delay the divorce process, and couples only go to these centers to goof off and shirk and they don't believe in them much.”) (39-year-old man, lawyer)

“Before, when a divorce was going to happen, the families of the man and the woman did their best to prevent the divorce from happening, but this is not the case now. Now no one respects their words, the young people do everything on their own, which is why the divorce rate is high” (37-year-old woman, PhD in demography)

### Inappropriate spouse choosing

Another reason for divorce among couples was inappropriate choice of spouse, that is, the couple had not chosen each other logically and properly at first. Another reason for divorce that was stated by the couple was a lack of proper counseling before marriage, as some participants stated that they got married without getting to know each other enough, which resulted in divorce. Some of these divorces were the result of forced marriages derived from customs or early marriage, which later led to divorce. Most of the forced and early marriages had taken place without women's desire and consent, which further led to dissatisfaction with the married life and ultimately led to divorce. In some marriages, the couples ignored class differences and cultural and social differences, which led to divorce. Also, in some cases, couples did not get to know each other much before marriage, and after the wedding, they realized how much they had different tastes. In some cases, too much emphasis on having a certain criterion on the part of the spouse, such as being rich or being a doctor, caused them to consider only having this criterion as enough for marriage, which led them to face many problems.

“I was not more than 15 years old when my father forced me to become my cousin's wife. From the beginning, I didn't like him very much. We had quarrels until we broke up” (17-year-old woman, diploma)

“I had a 15-year age difference with my wife. At first, I thought that because she was young, I could raise her however I wanted, but that didn't happen at all. We didn't understand each other very much, she made strange excuses.” (34-year-old man, junior high school)

“My husband was from Tehran. We met each other at university. We thought we could be happy together, but from the day of our wedding, cultural differences showed themselves. We were very different from each other. Our worlds were very different. He had expectations that were not acceptable to me. My family never accepted her as a son-in-law and we decided to separate and get divorced.” (33-year-old woman, bachelor's degree)

“Some couples don't think about cultural and class differences at all before marriage, but when they start living together, they see how much these differences can affect their lives. We had many cases that they got separated because of this class and cultural difference.” (41 years old woman, lawyer)

“I wanted to become a doctor, but I couldn't so I always dreamed of marrying a doctor. When I got in touch with my husband, even though I knew we weren't very compatible, but since he was a doctor, I tried to be exactly what he wanted. This didn't continue and our life lasted only two years, then we broke up.” (27-year-old woman, diploma)

“My husband's family had many problems. It was our fault that we didn't do much research before marriage. After the wedding, I realized that my father-in-law and one of my husband's brothers were drug addicts, and his mother was divorcing his father. Their problems affected our life a lot, they all involved us in their own problems. I had a lot of disagreements with my husband over these issues.” (22-year-old woman, diploma)

### Economic challenges

Another main reason for divorce that was frequently expressed by couples was economic challenges. Some couples had faced many financial problems due to bankruptcy or economic shocks such as the sudden increase in housing and car prices, which had led to tension in the family. Some men were imprisoned because of these financial problems and this issue led to divorce. Some women stated that their husband's unemployment and not having a suitable job were the reason for divorce. But the thing that caused the divorce between couples who were both employed was the separation of income and financial competition. Each of the couples saved separately for themselves, so in some cases, it caused competition between them, and this issue made them far from each other day by day and finally led to divorce. In general, due to the increase in economic inflation in the last decade and the increase in the cost of housing and other living expenses, Iranian families have suffered a lot of financial pressure, which has caused many problems in the lives of young couples.

“My husband was unemployed. I couldn't buy a coat once a year. My parents bought me a coat all the time. I was tired of this life, that's why I decided to get a divorce” (25-year-old woman, bachelor's degree)

“I sold my house and invested in the stock market. It was very good at first, but everything went wrong and I lost my capital totally. This was the beginning of all our problems. My wife kept making excuses and arguing with me, ‘why did you do this?’” (33-year-old man, master's degree)

“My wife and I were both working, but from the very beginning, my wife did not buy anything for the house. She used all her money to either buy gold or let her brother trade with it and give her a profit. We fought many times over this issue, it became such that we had determined what expenses each person should pay for the house, these tensions had increased so much that we stopped talking to each other at all.” (32-year-old man, PhD)

“One of the main reasons for divorce that I have seen is financial problems, most families are under pressure, and this financial pressure causes other problems such as violence and addiction, which ultimately lead to divorce.” (31-year-old woman, family counselor)

### Modeling of divorce

The growth of the divorce rate in the last decade has caused at least one divorce to take place in some Iranian families, which has caused a kind of imitation or fashion of divorce in society. Many couples take a model for divorce from their friends or relatives in some way. Also, the issue of divorce is always shown in the media, and this issue itself causes divorce to be no longer considered as an unfortunate event and becomes a custom or pattern.

“Until my sister got divorced, I didn't think about divorce at all, but when she got divorced, I was motivated to get a divorce. I said to myself, why should I stay in a life that doesn't bring me any pleasure, a few of my close friends had just gotten divorced.” (24-year-old woman, Associate Degree)

“Divorce has increased so much that most young people look at their friends and imitate them” (55-year-old man, bachelor's degree, divorcee's father)

“I was a child of divorce. I knew how many problems a child of divorce would face, that's why I kept telling myself that I would never get a divorce in my life, but it didn't go as I liked and I got divorced. I don't know, maybe my mother influenced my divorce.” (26-year-old woman, associate degree)

“Divorce has increased a lot in our society and it seems that it has become a custom and norm for everyone. Many people think of divorce more easily than before.” (49-year-old man, PhD in sociology)

1. **Family factors**

Another category was family factors, which were mostly related to events that happened within the framework of the family and led to tension and separation.

### Job’s impact on family life

One of these family factors was issues related to people's jobs that affected their family life. Some jobs led to husband and wife not being able to be with each other and this physical distance gradually separated them emotionally and caused dissatisfaction and separation. Also, having jobs such as driving a heavy car for men or nursing and having night shifts for women caused dissatisfaction in their spouses so that they had to choose between the work and the spouse. Also, some couples stated that their spouses worked in environments that were not safe for them, and their spouses could make mistakes and betray them, and this issue caused more tension and dissatisfaction with the spouse's job, which ultimately resulted in divorce.

“She was a nurse, she was in the hospital most of the time, she was on night shifts and this was very difficult for me. Every night I thought to myself that her colleague might approach her or that my wife might cheat on me. I asked her several times to stop working, but she refused.” (25-year-old man, bachelor's degree)

“My husband was a truck driver, sometimes he didn't come home for 20 days, well, it was very hard for me. I didn't have anyone here, my family was not with me, and I felt lonely all the time. I said to myself ‘What kind of husband do I have; he is never with me.’ We always fought about this issue and finally, we broke up.” (24-year-old woman, associate degree)

### Improper communication with spouse's family

Another reason for divorce was inappropriate communication with the spouse's family. Some participants stated that their spouse did not have a proper relationship with their family and disrespected them continuously, or they did not participate in mourning ceremonies and wedding celebrations, which caused dissatisfaction and tension, which ultimately resulted in divorce. Also, the inappropriate relationship with the spouse's family in some cases led to the interference of the families, and the life of the couple was affected. The involvement of families was seen more especially in couples who were younger.

“My husband always cursed my family, he always made fun of my brothers in front of me, and this bothered me a lot.” (26-year-old woman, associate degree)

“My wife cared about her family a lot, but she didn't pay attention to my family at all. Every time my family came home, she disrespected them. Because of this, we got into a lot of trouble and I divorced her.” (24-year-old man, bachelor's degree)

“My husband didn't accept my family. He kept saying I had no right to have a relationship with my sisters. Every time they invited us, he made an excuse and didn't want me to go. I was tired. My sisters and brothers were all complaining me about why I didn’t have a relationship with them.” (34-year-old woman, diploma)

### Accumulation of negative feelings in marital life

Some participants had some negative feelings such as hopelessness and fear of being abandoned in the future in their married lives, which led to the formation of unpleasant feelings and dissatisfaction with life, and finally resulted in divorce. Also, some other participants stated that they felt that their married life was unfair and this feeling of unfairness was more prevalent among women because they believed that they had given all their time to their husbands and children and had not been able to achieve their education or job and their dreams, and they have given up on their own interests, while their husbands had more time and were able to continue their studies or reach their work goals. Also, in some cases, some of the participants considered themselves to be at a higher level than their spouses in terms of education, beauty, etc., and this led to the formation of a feeling of superiority over them, which led to divorce.

“One of the reasons why I broke up with my husband was that I had no hope for him. He always left me alone in difficult situations, and this worried me about how I could rely on him.” (25-year-old woman, bachelor's degree)

“My wife and I both got a bachelor's degree together and we got married. I stayed at home and had a baby, but he took his master's degree and found a good job. Every time I wanted to take the master's exam, he prevented me. I felt that this life was not fair, that's why I broke up with him. And now I'm satisfied because I'm finishing my master's degree and I'll continue until I get a doctorate.” (34-year-old woman, bachelor's degree)

“My wife was not at my level, neither her appearance, nor her level of education, nor anything else, I was superior to her in all respects that was what everyone in the relatives said.” (28 years old, man, master degree)

### Issues related to children

Another reason for divorce was the challenges related to children. Some of the participants mentioned the main reason for their divorce as not having children or their spouse's unwillingness to have children, which caused disputes and separation. Also, the presence of a disabled child in the family caused many challenges for the couple, which later led to separation and divorce. Not having a son was another influential factor. In some traditional families, having a son is still preferable to having a daughter, and families want their child or grandchild to be a boy, and in cases where a woman does not give birth to a boy, she usually faces many challenges and is rejected by the husband's family. Although the sex of the baby is mostly determined by the father, but due to the lack of knowledge in traditional areas, there is a belief that it is the physical and sexual structure of the woman that determines the gender of the baby.

“My husband and I had been married for several years, but we didn't have children. I loved children, and the problem was on my husband's side, but he didn't accept to be treated, that's why I divorced her.” (30-year-old woman, diploma)

“I wanted to have two or three children while I was young, but my wife said that I never want to have children again. That's why we had a disagreement and we broke up.” (34 years old man, junior high school)

“Our first child was a boy, he was physically disabled, I cried all my life for 5 years, I thought of suicide many times, I wished for death every time I saw him, I told my husband to put him in a nursing home, but he didn't accept. I couldn't put up with anymore, so I filed for divorce.” (29-year-old woman, Bachelor's degree)

“My husband's family wanted a grandson from the beginning, but I gave birth to three daughters. They said that I was like my mother who only gave birth to girls. We don't have a brother; we are five sisters. They were bothering me a lot. They pressured me to accept that he would marry another woman.” (33-year-old woman, junior high school)

### Cheating on spouse

One of the reasons that some couples mentioned as the only reason for divorce was cheating, which was mentioned in both sexes. Some of the betrayals happened in the real world and had severe consequences, although these consequences were more for women and it was even possible that it would lead to their death. In some cases, which was expressed by many participants, it was virtual infidelity that is, having emotional relationships with the opposite sex in cyberspace, which led to tension and divorce. The spread of new social networks the access of the general population of Iran to it and the lack of sufficient training on how to use these networks have caused deviations in Iranian society, one of which is betrayal and cheating on the spouse.

“My husband didn’t have much commitment to me. I noticed several times that he was talking to other women, but every time I told myself that he was just talking until I realized that he was having sex with them. I couldn't take it anymore and got a divorce.” (33-year-old woman, bachelor's degree)

“I noticed that my wife is talking to a man on Telegram and emotional words are being exchanged between them. It was very difficult for me; our fight started from there.” (30-year-old man, bachelor's degree)

“Infidelity and cheating have increased, many of the people who go to the court are mostly for infidelity. In most of the cases I have seen, men cheat, but there are also women.” (48-year-old man, PhD in Sociology)

### Inefficient relationships

Another reason for divorce was ineffective communication the couple had not been able to establish a constructive relationship during their married life. In many cases, before the divorce happened, the couple had experienced some kind of emotional divorce and did not have much contact with each other and they only lived under the same roof. Some of the participants said that late in their married life, they did not talk to each other at all or did not try to provide sweet and good moments for each other. They said that because they felt that their life partner did not understand them, they talked less and refrained from expressing their feelings. In some cases, the married life was associated with secrecy and lying about various issues, and these issues have resulted in a kind of indifference towards marital duties.

“For the last year of our married life, we were completely separated and we didn't talk to each other at all. I asked several times to go on a trip together, maybe our life would get better, but he didn't accept it.” (25-year-old woman, bachelor's degree)

“My wife and I didn't understand each other at all. The best moments of my life were when my wife went to her father's house. It was hard for me to bear it. When she was home we slept separately, most of the time I slept in front of the TV in the living room.” (32-year-old man, PhD)

“My husband lied to me most of the time and if he did anything, he would hide it. When I asked him, he would say it was none of my business.” (33-year-old woman, bachelor's degree)

“My husband didn't care much that he has a wife and he should pay attention to her. It wasn't like that at first, but later his behavior changed so that he often came home so late that I fell asleep and he didn't see me.” (29-year-old woman, master's degree)

### Heterogeneity of lifestyle

In some cases, couples had different lifestyles, hobbies, and beliefs, and this issue caused them to behave in a completely different way in their married life. This had become a challenge. Also, most of them had different religious beliefs and they could not cope with these differences. Young couples did not understand each other in most of the important matters of life, such as lifestyle and clothing, life priorities and how to raise children, determining where to live, and even how to spend their free time. This lack of understanding was more in cases where couples were married without recognition and traditionally, and after marriage, they realized how different they were from each other.

“Some couples are opposite in terms of behavior and because they have not received any training to understand each other's differences, they face problems in life.” (37-year-old woman, PhD in Demography)

“My husband was very religious and he expected me to be like her. He said that you should pray in the early morning and wear a chador. I couldn't accept it at all.” (26-year-old woman, associate degree).

“My wife and I had no mutual understanding. We belonged to two different worlds. It was neither her fault nor my fault. She grew up one way and I grew up another way.” (33-year-old man, PhD)

“My husband said that we should definitely live in his city with his parents, but I couldn't do that at all. I wanted to live in a bigger city and we had the conditions to do it, but my husband was against it.” (25-year-old woman, bachelor's degree)

“I loved traveling and mountain climbing, but my wife was fond of going to the market and shopping, even when we went on a trip, we were all in the market and it was not pleasant for me at all. She was always looking for fashion and clothes while I didn't care at all.” (Man, 28 years old, Master's degree)

### Violence

Another reason for divorce was violence, which was mostly mentioned by women as a reason for divorce. Some of the participants stated that they faced all kinds of verbal, physical, sexual, etc. violence from their spouses, and their spouses insulted and humiliated them in family gatherings. Of course, in some cases, this violence was mentioned in a milder form and in terms of verbal and psychological violence by men as a reason for divorce. In general, violence by men was mostly used in the form of physical violence, and the violence used by women was more verbal and psychological.

“Whenever my husband got nervous and angry, he started swearing and beating me.” (19-year-old woman, junior high school)

“My husband never knew how to treat me, even in sex, instead of trying to give me pleasure, he bothered me more, so that I often did not want him to get close to me.” (33-year-old woman, bachelor's degree)

“My wife used to be a source of embarrassment and she used to talk to me in public in such a way that everyone would blame me that why I let my wife disrespect me like that.” (24-year-old man, bachelor's degree)

### Inability to manage challenges

Another reason for divorce was the lack of a culture of dialogue between couples and appropriae strategies to resolve conflicts. So, most of the time, the smallest issue turned into a continuous and long-term argument between the couple and caused anger and discomfort between them. In some cases, instead of solving these conflicts, couples took revenge and showed this revenge in different ways. In some cases, the spouses involved the families in tension with the smallest tension that happened and caused them to interfere and continue the tension. The presence of stubbornness and hasty decision-making were among the other cases that, from the point of view of most key informants, were grounds for divorce.

“Every time there was a problem, we couldn't convince each other, it continued and sometimes it led to long-term anger and quarrel.” (21-year-old woman, diploma)

“Every time I argued with my husband, he would try to take revenge on me somehow. He would annoy me with his behavior. He didn't care if our problem was solved, he thought I was annoying on purpose.” (27-year-old woman, associate degree)

“As soon as a small fight happened between me and my wife, she used to tell her family immediately, and this would make the case take longer. This behavior was very bad because instead of helping, her family caused more problems.” (33-year-old man, PhD)

### Core category: Weakening of the family institution in the transition from tradition to modernity

In-depth analysis of the interview data revealed that although divorce among young couples in western Iran is influenced by three categories of individual, socio-cultural, and family factors, these three categories are ultimately unified under a single main category: the weakening of the family institution in the transition from tradition to modernity. This main category serves as a conceptual umbrella encompassing all subcategories and extracted codes. In other words, what ultimately emerged from the participants’ interviews is that divorce in the studied society is not merely the result of isolated individual problems or scattered economic and social pressures, but rather a reflection of a deeper structural transformation. On the one hand, traditional family support systems, such as the role of elders, constructive family intervention, and the strong taboo against divorce, are weakening and eroding. On the other hand, modern adaptive mechanisms, such as efficient counseling centers, communication and conflict resolution skills, and formal institutional supports, have not yet been fully developed or lack the necessary effectiveness. This gap and tension between the traditional and modern systems provides the main context for the occurrence of divorce among young couples. Concrete evidence of this main category can be observed throughout the subcategories. In the individual factors category, subcategories such as individualism, positive understanding of divorce consequences, and having defective premarital relationships indicate the rise of modern values emphasizing personal autonomy over collective and family commitments. In the socio-cultural factors category, subcategories such as de-tabooing of divorce, women-oriented social changes, media and social networks, and modeling of divorce reflect the shift of cultural norms away from traditional values without the replacement of new established norms. In the family factors category, subcategories such as improper communication with the spouse's family, accumulation of negative feelings, heterogeneity of lifestyle, and inability to manage challenges show that neither the traditional system, due to reduced effective family intervention, nor the modern system, due to the lack of communication and conflict resolution skills, is capable of repairing marital ruptures. Therefore, the main category, the weakening of the family institution in the transition from tradition to modernity, effectively explains this intermediate situation in which young couples have lost traditional supports while lacking the modern tools to maintain their married life, and this structural void pushes them toward divorce.

## Discussion

This study was conducted with the aim of explaining the reasons for divorce among young couples in the west of Iran. Results showed that divorce is a complex and multidimensional phenomenon that is influenced by various individual, socio-cultural, and family factors.

Sexual dissatisfaction was one of the individual factors underlying divorce, which was consistent with previous research [8,26]. In the study by Daneshfar & Keramat (2023), weakness in sexual performance and sexual dysfunction were reported as one of the main reasons for divorce [30]. In the current research, a part of sexual dissatisfaction was caused by sexual problems and the most important part was the couple's misconceptions about sexual relations, which were often formed by watching porn movies and comparing their sexual relations with them, which caused them to have inappropriate expectations from their sexual partners, which ultimately led to sexual dissatisfaction resulting in a divorce.

Psychological disorders were another reason for divorce, which included a wide range of mental problems such as bipolar disorder, severe obsession, depression, anxiety disorders, schizophrenia, and paranoid personality disorder which was one of the interesting findings in this study and added to previous studies that the relationship between divorce and mental disorders can be two-way, that is, on the one hand, mental disorders can ultimately lead to divorce, and on the other hand, according to most of the studies Divorce can lead to mental disorders in divorced couples, especially women [31,32]. However, the problem that existed in the studied areas and may be different from other countries was the individual's resistance to accepting the illness and going to a counselor. In fact, due to the taboos about visiting a psychologist in the studied areas, most couples were reluctant to go to a psychologist for the treatment of these diseases, and this problem caused the disease to worsen day by day, making it difficult for their partner to continue the married life.

Another finding was the spouse's behavioral characteristics, which was the basis for divorce. Some of these characteristics, such as nervousness weak ability to express love, and fanaticism were more common among men, which can be formed from patriarchy and gender beliefs in the society. Because expressing affection and love in the studied society is considered more of a feminine act and men try to express their love and affection less and show it more with their behavior, this can cause problems in cases where their wives are more emotional and need attention and receiving emotional words.

Individualism was another determinant of divorce and it was one of the new and interesting findings in this research; one of the couples thinks more about their well-being and their comfort and enjoyment rather than feeling more responsible for their spouse and family and spending more time with their family, and this issue cause them to face problems in the family. In the studied society, which is a traditional society, the introduction of new communication technologies and the formation of a new lifestyle has caused the level of individualism to increase, and this can cause problems for the families that were formed in that society.

Physical and appearance problems were another reason for divorce. Infertility was one of the main physical problems that led to divorce, which was in line with previous research [33,34]. An interesting finding was that infertility affected women more because it is based on popular cultural beliefs in the region. Infertility is considered more of a women's problem and it is difficult for men to accept this problem. Another reason for divorce was physical disability, most of which occurred after marriage and due to accidents such as driving and work injuries; the physical disorder made it difficult for their partner to continue their married life. Being fat and not being good-looking was another reason for divorce while being fat was considered one of the criteria of beauty until a few decades ago. Due to the introduction of modern technologies and familiarity with fashion in the world, the lifestyle and the society's attitude towards obesity changed and it was considered as an ugliness criterion rather than as a measure of beauty.

Another reason for divorce was the formation of a positive understanding of the consequences of divorce, which, although it seems to be more of an individual factor, is rooted in the changes that have taken place in society and have changed the view of young people on the phenomenon of divorce. In the past, it was considered as an ominous phenomenon, but now it is considered as a way to escape from the unhappy married life because couples believe that after divorce they can build their lives again and they refer to it as a kind of rebirth. Also, some women look at it as a source of income and dowry, and in this way, they can earn a good income for themselves, although getting a dowry is a long-term process and may take years.

Having defective relationships before marriage was one of the other factors that led to divorce, which was consistent with previous studies [35]. Smith & Wolfinger's (2023) study also showed that having polysexual relationships before marriage has a strong relationship with divorce [36]. Relationships before marriage can affect the quality of relationships after marriage. People who have been involved in relationships before marriage have suffered a lot of psychological damage, these relationships affect their married life. In some cases, multiple relationships before marriage lead to sexual diversity seeking after marriage, which leads to infidelity and other harms, and ultimately results in divorce.

Another reason that led to divorce was drug use, which has always been mentioned as one of the most important reasons for divorce in previous studies [8,37]. However, the noteworthy point in this study was that most of the participants who got divorced due to the use of drugs had spouses who were addicted to industrial drugs, and these industrial drugs caused them to show risky behaviors that threatened their partner's health, that's why the divorce happened.

Socio-cultural factors were other factors that led to divorce. De-tabooing of divorce was one of these socio-cultural factors. Until a few decades ago, divorce was a taboo in Iranian society, and few families were willing to allow divorce to happen in that family. The burden of this taboo was heavier for women, and women could not lead a normal life after divorce and faced social stigma, but in the recent decade, a lot of changes happened in this field, and the taboo of divorce was reduced to a great extent. This issue caused many couples to think about and act on divorce much easier than in the past. This change of attitude towards divorce caused even divorced people to be no longer under social pressure as before and can return to normal life and remarry more easily than before.

Among the other reasons for divorce, which were mostly related to women, were women-centered social changes. In the last two or three decades, women in Iran were able to change their economic and social status by entering higher education and taking up jobs. They no longer depended on men to continue their lives as in the past, and they had more independence. This made it easier for them to take action for divorce. The study by Vignoli et. al. (2018) also reported women's employment as one of the facilitators of divorce in Italy and Poland [38]. However, it has been reported in some studies that the level of education of men leads to an increase in satisfaction with married life [39]. In societies where divorce has many negative consequences for women and their families, women's education and employment can lead to independence and reduce their dependence on the family so that they can make decisions on divorce more easily. In Iran, entering university and higher education made women more aware of their rights, and this made them more resistant to men's coercion in the family and confront them; one of the forms of resistance and confrontation was divorce and separation.

The media and social networks were other factors that contributed to the divorce, which was under study by Clayton 2014 [40]. In the study of Liu et al 2023, which was conducted in China, it was reported that access to the Internet and presence in social networks are related to the increase in divorce [41]. With the rapid growth of social networks and access of Iranian people to them in the last decade, these media have become an inseparable part of the lives of Iranians, so they spend many hours of their time daily on these networks. For this reason, these networks have created many changes in people's social relationships and lifestyles, which may not be acceptable to the culture of traditional societies, that's why they create tension and conflict. Another part of the impact of social networks on people's lives is that they compare their lives with bloggers and celebrities resulting in disappointment and dissatisfaction with married life, and in the long run it has destructive effects because bloggers only show the positive aspects of their lives exaggeratedly.

The lack of a suitable support network was another social factor underlying the divorce. Until a few decades ago, families were responsible for solving most of the couples’ family disputes and they gave all their support to the couples so that their conflict was resolved and did not lead to separation, but today this role is no longer the responsibility of the families and they are entrusted to the related centers. Unfortunately, these centers are unable to play this role well due to the limited access and the problems they have, which is why the rate of divorce in society is increasing day by day.

Another socio-cultural determinant of divorce was choosing an inappropriate spouse. In the study of Barikani et al 2012, the inappropriate choice was one of the main reasons for divorce in Iranian couples [42]. The way of choosing a spouse and the process of choosing a spouse can greatly affect the quality of life together. Early and forced marriages always have a higher risk of divorce [37,43]. Also, not considering the social and cultural differences in marriage can be a problem after the wedding, and if the couple cannot manage this issue, they may have tension. A person's purpose and reason for marriage can also influence the choice of the desired person, and if a person does not have the proper motivation and criteria for marriage, he may not make the right choice and face problems in life.

Economic challenges were another reason for couples to divorce, which is in line with previous research [26,27]. In Sadeghi & Agadjanian's 2019 research, the economic insecurity of the family was one of the variables influencing couples’ willingness to divorce [44]. Some of these economic challenges were related to financial problems, low income, and lack of housing, which is partly related to the growth of inflation in Iran in the last decade. Another part of these financial challenges was the financial competition of couples with each other and separating their incomes, which ultimately led to conflict in the family resulting in separation. This issue is especially noticeable in couples where both are employed. This finding added to previous studies that proper management of family income, especially when both spouses are employed, can be effective in preventing tension and divorce.

Another finding was the modeling of divorce, which showed that the prevalence of divorce in society and its frequent observation causes a kind of imitation and modeling of divorce, which can be effective in people's decision to divorce. Previous studies have also shown that the risk of divorce is higher among those who have experienced the divorce of their parents or close friends [35,45].

The effects of job on family life was one of the family factors of divorce, which was related to the negative effects that the job of one of the spouses had on their lives, which was consistent with previous research conducted in this field [35,44]. Some couples considered their partner's work environment to be unsafe and inappropriate, and this caused their dissatisfaction. Also, the job status of some participants was such that they had to be away from their families for many hours, and this issue was unpleasant for their partners and led to conflicts.

Inappropriate communication with the spouse's family was another reason for divorce. In the studied society, the traditional structure of families is still preserved and the families are constantly in contact with each other. Thus, if one of the spouses does not have much desire to communicate with their partner's family, it is unpleasant for the family and they put pressure on the spouse. Also, some families interfere in the lives of young couples under the pretext of supporting them and raising more challenges. In the research of Qamar & Faizan 2021 in Pakistan, the involvement of families is mentioned as one of the most important reasons for divorce [27].

Accumulation of negative feelings was another reason for divorce. In the research of Qamar & Faizan 2021 in Pakistan, the feeling of disappointment in married life was one of the determinants of divorce [27]. Some couples in had faced feelings in their married life that discouraged them from continuing and caused them to decide to get a divorce. Some of the participants felt fear and despair about the future and felt that married life had limited them in reaching their personal goals and dreams. Also, the existence of a feeling of superiority over the spouse in one of the couples was not without influence in the occurrence of this disappointment, which was mostly rooted in the way they chose. In the study of Jalili et al 2017, which was conducted in Iran, the feeling of inferiority in married life was one of the determinants of divorce [46]. Also, the feeling of inequality and unfairness of married life was more common among women, because women believed that they spent more time on family and raising children than their husbands, and this problem has caused them to not be able to continue their studies or jobs. In a research conducted in Spain, the feeling of unfairness and inequality in the division of household duties among women is mentioned as one of the reasons for divorce [47].

Another finding was the issues related to children. According to the traditional structure of the studied society, having a child is always considered one of the reasons for marriage, and if having children in a marriage faces problems, it may lead to divorce. The findings of the Naab et al 2019 research in Nigeria also showed that female infertility is one of the reasons for divorce [48]. It was also reported in the Manhart & Fehring et al 2023 research that the continuous use of contraceptives can increase the risk of divorce [49]. In a research conducted in Iran in 2023, a significant relationship was found between the stress caused by infertility and emotional divorce among both spouses [33]. Due to the existence of patriarchal structures in the studied society, having a male child is preferable to a female child in some families. For this reason, women who give birth to girls are under pressure from their husbands and their husband's families, and it is possible that their husbands are under pressure to divorce them or they agree that their husbands marry another woman so that they can have a son. Having a disabled child also causes a lot of social and family challenges. Some people are unable to take care of them and think about divorce. The findings of Anchesi et al 2023 also showed that having a child with autism increases the likelihood of divorce in the family [50]. In the research of Yoosefi Lebni et al 2021, it was also reported that mothers of disabled children face many problems and dissatisfaction in married life [51].

Another family factor underlying divorce was infidelity and cheating, which has been mentioned in previous studies in different countries as one of the important determinants of divorce [26,52]. In the study of Rinaldo et al 2024 in Indonesia, infidelity was reported as one of the main reasons for divorce [14]. In the present study, the type of encounter with this issue is different in men and women who are victims of infidelity. When a woman commits infidelity, it is more likely to lead to divorce or even to her death because in the studied society, men's cheating is more acceptable than women's cheating, when men cheat, in many cases, women accept it unless this cheating is repeated continuously.

Inefficient relationships were another factor behind divorce, which was consistent with previous research [37]. In a study in Saudi Arabia, lack of expression of emotions and feelings had a significant relationship with emotional divorce between spouses [53]. Young couples did not have a stable relationship while living together, and before their lives reached the stage of divorce, they had experienced a kind of emotional divorce, so that they only lived under one roof and did not have a constructive relationship with each other, and they rarely talked or traveled together. Also, in some cases, this non-productive relationship was accompanied by lies and concealment about various issues which added to the dissatisfaction of the couple.

Another reason for divorce was heterogeneity in lifestyle, which was following Qamar & Faizan's 2021 research in Pakistan (26). Differences in personality traits and lifestyle were also reported as one of the main reasons for divorce among Hmong women in Her & Xiong's 2023 research [54]. Also, the lack of mutual understanding in the married life, which mostly stems from the couple's lack of knowledge of each other before marriage and ignoring the differences, was another reason for divorce. In many cases, couples had not learned the ability to get along and manage the differences they had with their spouses, and this issue led to tension in their lives.

Violence was one of the main reasons for divorce, which is in line with most previous studies [8,55]. Also mentioned is violence as one of the reasons for divorce in Shita & Zeleke's 2024 research [1]. In the present study, violence in the family is mostly done by men, and women are the only victims of violence, which is related to the patriarchal atmosphere ruling the society.

The inability to manage challenges was another finding that showed that couples had not learned how to manage conflicts in their lives, and the smallest problem that occurred in their lives continued and turned into a big challenge. In the research of Nakhaee et al 2020, the inability to resolve marital conflicts is mentioned as one of the most important reasons for divorce [23].

### Limitations and strengths of the study

One of the strengths of this research was that, unlike most other research that only examined women's point of view, in this research, in addition to women, men and also key informants who had valuable and comprehensive opinions on the phenomenon of divorce were interviewed. This resulted in deepening the understanding of the phenomenon of divorce, which can be the basis for subsequent social and cultural interventions to reduce divorce. However, this research had some limitations, one of these limitations was the concern of the participants about the publication of the recorded files, which made them reluctant to participate in the research at first. But the researcher explained the process of participating in the research and publishing the findings, as well as ensuring that the recording files won't be in anyone else's possession and got their consent. Another limitation of the research was that some of the participants were women, and to be able to share their experiences more easily with the researcher, it was necessary to use a female researcher for the interviews.

## Conclusion

The results of this study showed that divorce is a complex and multi-dimensional phenomenon influenced by individual, socio-cultural, and family factors. However, beyond these three categories, the findings were ultimately unified under a single core category: the weakening of the family institution in the transition from tradition to modernity. This core category reflects a deeper structural reality in which young couples in western Iran find themselves caught between two worlds. On the one hand, traditional support systems, including family mediation, the role of elders, and the social taboo against divorce, have significantly weakened. On the other hand, modern adaptive mechanisms such as efficient counseling centers, communication skills, and formal institutional supports have not yet been fully established or are not sufficiently accessible. This gap and tension between tradition and modernity creates a structural void that pushes young couples toward divorce. In other words, divorce in the studied context is not merely the result of individual failures or isolated economic problems, but rather a reflection of a broader social transition in which the family institution has lost its traditional resilience without gaining modern stability. Therefore, addressing the phenomenon of divorce requires understanding this fundamental transformation and developing interventions that bridge the gap between traditional and modern support systems.

At the individual level, the rate of divorce can be reduced by educating couples about sex and making them aware of sexual issues, holding multiple and coherent communication skills workshops for couples before and after marriage, preventing superficial and unrecognized marriages, and training the awareness for the roles of husband and wife and strengthening the culture of responsibility in the married life. Also, at the family and social level, divorce can be prevented to some extent by creating a culture to eliminate the stigma of referring to a psychologist, training to accept the differences in the personality and lifestyle of couples and how to manage these differences, training families on how to manage conflict and compromise among young people, education and culture building in the use of social networks and managing relationships in them, paving the way to refer to mental health centers for the treatment of mental illnesses, empowering couples after marriage, providing educational packages for choosing a spouse and referring couples to counseling centers before marriage.
